# Comparison of long-term oncologic outcomes of central lumpectomy and conventional breast-conserving surgery for invasive breast cancer: propensity score matching analysis

**DOI:** 10.1007/s10549-024-07297-8

**Published:** 2024-03-25

**Authors:** Ji-Jung Jung, Jong-Ho Cheun, Hong-Kyu Kim, Han-Byoel Lee, Hyeong-Gon Moon, Ki-Tae Hwang, Wonshik Han

**Affiliations:** 1 Department of Surgery, Seoul National University College of Medicine, 101 Daehak-ro, Jongno-gu, Seoul, 03080 Republic of Korea; 2 Department of Surgery, Seoul Metropolitan Government Seoul National University Boramae Medical Center, Seoul, Republic of Korea; 3 Biomedical Research Institute, Seoul National University Hospital, Seoul, Republic of Korea; 4 Cancer Research Institute, Seoul National University, Seoul, Republic of Korea

**Keywords:** Breast cancer, Breast-conserving surgery, Oncologic safety

## Abstract

**Purpose:**

Central lumpectomy (CL) is a breast-conserving surgical (BCS) technique that involves excision of the nipple-areolar complex with breast tumor in centrally located breast cancers. We aimed to investigate the long-term clinical outcomes of CL in comparison with conventional BCS (cBCS).

**Methods:**

Patient records who underwent BCS with clear resection margins for invasive breast cancer between 2004 and 2018 were retrospectively reviewed. Of the total 6,533 patients, 106 (1.6%) underwent CL. Median follow-up duration was 73.4 months. 1:3 propensity score matching (PSM) and inverse probability of treatment weighting (IPTW) were used to minimize selection bias.

**Results:**

The CL group showed a significantly higher ipsilateral breast tumor recurrence (IBTR) rate than the cBCS group (10-year IBTR rate: 5.8% vs. 3.1%, *p* = 0.004), even after adjusting for other variables (hazard ratio (HR), 2.65; 95% confidence interval (CI), 1.07–6.60, *p* = 0.048). However, there were no significant differences observed in regional recurrence, distant metastasis, or overall survival rates between the two groups. Both PSM and IPTW analyses showed significantly higher IBTR in the CL group (PSM HR, 3.27; 95% CI, 0.94–11.36; *p* = 0.048 and IPTW HR, 4.66; 95%CI, 1.85–11.77; *p* < 0.001). Lastly, when analyzing 2,213 patients whose tumors were located within 3 cm of the nipple, the CL group showed a significantly higher IBTR than the cBCS group before and after PSM.

**Conclusion:**

CL was associated with a higher rate of IBTR compared to cBCS, while other survival outcomes were comparable. For centrally located tumors, CL may be considered for patients preferring breast preservation. However, higher risk for IBTR should be informed and careful surveillance may be necessary during the early post-operative follow-up periods.

**Supplementary Information:**

The online version contains supplementary material available at 10.1007/s10549-024-07297-8.

## Introduction

Breast-conserving surgery (BCS) is a well-established and preferred treatment for the majority of patients with early breast cancer. Combined with radiation, BCS can offer patients better cosmetic satisfaction and equivalent oncologic outcomes compared to mastectomy [1]. In contrast, mastectomy is preferred for centrally located breast cancer (CLBC) due to concern for oncologic safety regarding tumor involvement of the nipple-areolar complex (NAC) and failure to achieve satisfactory cosmetic outcomes due to resection of the NAC. Further, a previous study has shown that BCS with preservation of the NAC is feasible only for strictly selected patients [2].

Central lumpectomy (CL) is a BCS technique that excises NAC with breast tumors. Considering that breast conservation is associated with improved patient satisfaction compared to mastectomy, CL is a reasonable surgical option for patients who strongly wish to conserve their breasts [3]. Several studies have reported superior satisfaction and quality of life following BCS compared to mastectomy and/or reconstruction [3, 4]. It is also known that centrally located breast tumors are not an absolute contraindication for BCS [5].

Several studies have investigated the survival of patients undergoing CL [6, 7]. A recent study using the SEER database reported a comparable overall survival rate of BCS for CLBC compared to mastectomy [7]. Information regarding the rate of local recurrence following CL, however, is limited. Another SEER study compared the oncologic safety of BCS for CLBC with that of BCS for non-CLBC and showed comparable oncologic outcomes in terms of 5-year local recurrence-free survival. The analysis, however, was confined to stage I and II breast cancers [6]. Recently, neoadjuvant chemotherapy has led to an increased rate of BCS [8], suggesting that more advanced-stage patients are eligible for CL. Consequently, the oncologic outcomes of CL warrant reassessment to reflect the recent shift toward increased adoption of BCS.

This study investigates the long-term clinical outcomes of CL compared to conventional BCS by PSM to reduce confounding and minimize selection bias. We also evaluate tumor-to-nipple distance to identify confounders based on tumor location.

## Methods

This study was approved by the Institutional Review Board (IRB) of Seoul National University Hospital (IRB No. H2209-011–1355). The Declaration of Helsinki and good clinical practice guidelines were followed, and the need for informed consent was waived.

### Study design

We retrospectively obtained baseline clinicopathological data of patients who underwent BCS for invasive breast cancer between January 2004 and December 2018 from the database of the Seoul National University Hospital Breast Care Center. We excluded patients with stage IV breast cancer, bilateral breast cancer, male breast cancer, recurrent breast cancer, or synchronous or metachronous cancer in other organs. Bilateral breast cancer was excluded due to higher risk of recurrence and likelihood of carrying BRCA mutations. As the involved resection margin was strongly associated with IBTR [9], our study only included patients with clear margins, defined as “no ink on tumor.” The initial breast cancer was clinically or pathologically staged according to the 7th American Joint Committee on Cancer staging criteria. The distance from the nipple was collected from preoperative magnetic resonance imaging and breast sonography reports. Hormone receptor status, including estrogen and/or progesterone receptors, was defined as positive when dyed > 1% on immunohistochemistry. Human epidermal growth factor receptor type 2 (HER2) status was assessed using anti-HER2 antibodies and/or fluorescence in situ hybridization. A Ki-67 level with positivity of < 10% was defined as low according to a previous study conducted in our institution [10].

### Surgical methods for breast conservation

CL was considered when the tumor was located close to the nipple on breast radiologic exams including breast sonography and breast MRI and when nipple excision was deemed inevitable. All patients with suspected invasion to the nipple were given the choice of whether to receive CL or mastectomy and shared decision-making was made by providing detailed description of surgical procedures. Nonetheless, the final decision leading up to CL primarily relied on the surgeon’s discretion due to cultural preference for passive acceptance of the doctor’s care. Most patients who underwent CL were approached through an elliptical incision or circumareolar incision (supplementary fig.S1). Besides, there was no patient who voluntarily selected CL although nipple conservation was possible. Notably, two patients whose tumors were located far from the nipple but underwent NAC resection for the purpose of oncoplastic surgery were excluded from the current study. The conventional BCS group only included patients who underwent oncoplastic surgery with NAC preserved. The choice of surgical methods did not affect the subsequent radiotherapy treatment regimen or fractionation schedules.

### Pathologic process for resection margin assessment

Following lumpectomy, all six margins of the excised mass were marked with distinct ink colors. Subsequently, the specimen was sliced at 5-mm intervals. Final margin was considered negative/clear when no tumor cell touched the ink on any of the six surfaces of the lumpectomy specimen.

### Recurrence and recurrence-free survival

Ipsilateral breast tumor recurrence (IBTR), the primary endpoint of the current study, was defined as the first recurrence in any quadrant of the ipsilateral breast. Regional recurrence was defined as recurrence in the ipsilateral regional lymph nodes, including the axillary, supraclavicular, infraclavicular, and internal mammary nodes. Recurrence-free survival was defined as the time interval between the date of surgery and pathologic or radiologic confirmation of recurrence. Regarding competing risks, other types of recurrence, including regional recurrence or distant metastasis prior to IBTR, were treated as censored events when calculating the IBTR-free survival rate. Overall survival (OS) was defined as the interval from surgery to death or last follow-up.

### Statistical analysis

Categorical variables and continuous variables were compared using Pearson’s χ^2^ test and one-way analysis of variance, respectively. The survival curves were derived using the Kaplan**–**Meier method, and the difference was analyzed using the log-rank test. The Cox proportional hazard regression model was used to estimate the adjusted hazard ratio and adjust for variables associated with the recurrence rate. Using Cox regression analysis, we adjusted for clinicopathological variables affecting IBTR, such as age at operation, tumor stage, histologic grade, lymphovascular invasion, hormone receptor status, HER2 status, Ki-67 level, and adjuvant treatment. Further, to minimize potential selection bias between the two groups, we conducted 1:3 PSM, including age at operation, year of surgery, tumor stage, histologic grade, lymphovascular invasion, hormone receptor status, HER2 status, Ki-67 level, and administration of neoadjuvant and adjuvant treatments. Estimated propensity score was further used to conduct inverse probability of treatment weighting (IPTW) analysis. Statistical significance was set at *p* < 0.05. All analyses were performed using SPSS (version 27.0; SPSS, Inc., IBM, Armonk, USA), and figures were plotted using GraphPad Prism™ (version 9.0; GraphPad Software, San Diego, CA, USA). PSM was conducted with “MatchIt” R package (version 3.6.3) [11].

## Results

### Patient demographics and characteristics

A total of 6,533 patients were identified who underwent BCS and met our inclusion criteria. For all patients, mean age at operation was 50.0 ± 9.8 years (range, 19.0–88.0). Most patients had hormone receptor-positive (73.3%) and/or HER2-negative (83.5%) tumors. Neoadjuvant chemotherapy was administered to 1,243 patients (19.0%) and 6,187 patients (94.7%) received adjuvant radiotherapy. The clinicopathological characteristics of the patients are summarized in Table 1.

**Table 1:** Clinical characteristics of all patients according to the operation method

Characteristics	All patients (*n* = 6,533)	Central lumpectomy (*n* = 106)	Conventional BCS (*n* = 6,427)	*p*-value
Age at operation (years)^*^	50.0 ± 9.8	51.1 ± 9.5	49.9 ± 9.9	0.231
< 50	3319 (50.8%)	46 (43.4%)	3273 (50.9%)	0.124
≧ 50	3214 (49.2%)	60 (56.6%)	3154 (49.1%)	
Year of surgery				
2004–2012	3057 (46.8%)	41 (38.7%)	3016 (46.9%)	0.091
2013–2018	3476 (53.2%)	65 (61.3%)	3411 (53.1%)	
T stage^†^				
T1	3609 (55.2%)	66 (62.3%)	3543 (55.1%)	0.328
T2	2664 (40.8%)	37 (34.9%)	2627 (40.9%)	
T3–4	260 (4.0%)	3 (2.8%)	257 (4.0%)	
N stage^†^				
N0	4173 (63.9%)	66 (62.3%)	4107 (63.9%)	0.206
N1	1624 (24.9%)	32 (30.2%)	1592 (24.8%)	
N2–3	716 (11.0%)	7 (6.6%)	709 (11.0%)	
Unknown	20 (0.3%)	1 (0.9%)	19 (0.3%)	
Histologic grade				
I–II	3935 (60.2%)	61 (57.5%)	3874 (60.3%)	0.923
III	2594 (39.7%)	41 (38.7%)	2553 (39.7%)	
Unknown	4 (0.1%)	4 (3.8%)	0 (0.0%)	
Lymphovascular invasion				
Present	1691 (25.9%)	36 (34.0%)	1655 (25.8%)	0.061
Absent	4673 (71.5%)	68 (64.2%)	4605 (71.7%)	
Unknown	169 (2.6%)	2 (1.9%)	167 (2.6%)	
Hormone receptor status				
Positive	4791 (73.3%)	86 (81.1%)	4705 (73.2%)	0.067
Negative	1742 (26.7%)	20 (18.9%)	1722 (26.8%)	
HER2 receptor status				
Positive	1079 (16.5%)	28 (26.4%)	1051 (16.4%)	0.006
Negative	5454 (83.5%)	78 (73.6%)	5376 (83.6%)	
Ki-67 index				
< 10%	4718 (72.2%)	75 (70.8%)	4643 (72.2%)	0.795
≧ 10%	1785 (27.3%)	30 (28.3%)	1755 (27.3%)	
Unknown	30 (0.5%)	1 (0.9%)	29 (0.5%)	
Neoadjuvant CTx				
Administered	1243 (19.0%)	14 (13.2%)	1229 (19.1%)	0.124
Not administered	5290 (81.0%)	92 (86.8%)	5198 (80.9%)	
Adjuvant CTx				
Administered	3272 (50.1%)	61 (57.5%)	3211 (50.0%)	0.124
Not administered	3255 (49.8%)	45 (42.5%)	3210 (49.9%)	
Unknown	6 (0.1%)	0 (0.0%)	6 (0.1%)	
Adjuvant RTx				
Administered	6187 (94.7%)	96 (90.6%)	6091 (94.8%)	0.068
Not administered	330 (5.1%)	10 (9.4%)	320 (5.0%)	
Unknown	16 (0.2%)	0 (0.0%)	16 (0.2%)	
Adjuvant HTx				
Administered	4712 (72.1%)	81 (76.4%)	4631 (72.1%)	0.343
Not administered	1803 (27.6%)	25 (23.6%)	1778 (27.7%)	
Unknown	18 (0.3%)	0 (0.0%)	18 (0.3%)	
HER2-targeted treatment				
Administered	783 (12.0%)	18 (17.0%)	765 (11.9%)	0.110
Not administered	5750 (88.0%)	88 (83.0%)	5662 (88.1%)	

*BCS* breast-conserving surgery, *HER2* human epidermal growth factor receptor-2, *CTx* chemotherapy, *RTx* radiotherapy, *HT*x hormone treatment

^*^Values are means ± standard deviation

^†^Stratified according to the American Joint Committee on Cancer (AJCC) 7th TNM stage, patients who underwent neoadjuvant CTx were evaluated with clinical stage

Overall, 106 (1.6%) patients underwent CL, with the remaining 6,427 (98.4%) undergoing conventional BCS. Compared to the conventional BCS group, patients in the CL group underwent surgery more recently and showed more lymphovascular invasion, hormone receptor positivity, and HER2 positivity. Neoadjuvant chemotherapy and radiotherapy were also administered more frequently in the conventional BCS group compared to the CL group (Table 1).

### Impact of surgical technique on survival outcomes

**Fig. 1:**
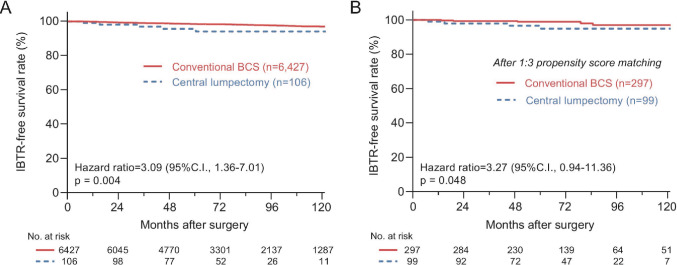
Kaplan–Meier curves for ipsilateral breast tumor recurrence-free survival comparing the surgical methods. The Kaplan**–**Meier curves show the differences in IBTR between the two groups before (A) and after (B) 1:3 propensity score matching. P-values were calculated using the log-rank test and hazard ratios were calculated using the Cox regression test. *BCS* breast-conserving surgery, *CI* confidence interval, *IBTR* ipsilateral breast tumor recurrence

During the median follow-up period of 73.4 months, six (5.7%) and 134 (2.1%) IBTR events were noted in the CL and conventional BCS groups, respectively. The CL group had significantly higher IBTR than the conventional BCS group (10-year IBTR: 5.8% vs. 3.1%; hazard ratio [HR], 3.09; 95% confidence interval [CI], 1.36–7.01; Log-rank *p* = 0.004) (Fig. 1A). In contrast, RR-free survival, distant metastasis (DM)-free survival (DMFS), and OS rates were not significantly different between the two groups (Supplementary Fig. S2).

After adjusting for clinicopathological variables, surgical strategy for lumpectomy remained a significant prognostic variable associated with IBTR (HR, 2.65; 95%CI, 1.07–6.60, *p* = 0.048) (Table 2). In addition, younger age, higher histologic grade, lymphovascular invasion, no adjuvant chemotherapy, and no radiotherapy were associated with higher IBTR (*p* < 0.001).

**Table 2:** Log-rank and Cox regression analyses for ipsilateral breast tumor recurrence-free survival

Characteristics	Log-rank analysis		Cox regression analysis	
	Hazard ratio [95% CI]	*p*-value	Hazard ratio [95% CI]	*p*-value
Age at operation (years)				
< 50	Ref.	0.012^†^	Ref.	0.003
≧ 50	0.64 [0.45–0.91]		0.57 [0.39–0.83]	
Year of surgery				
2004–2012	Ref.	0.683	–	–
2013–2018	1.08 [0.74–1.58]			
T stage^*^				
T1	Ref.	0.001^†^	Ref.	0.758
T2	1.42 [1.00–2.00]		1.05 [0.69–1.59]	
T3–4	2.87 [1.55–5.32]		1.34 [0.61–2.94]	
N stage^*^				
N0	Ref.	0.068^†^	Ref.	0.791
N1–3	1.38 [0.99–1.93]		0.94 [0.60–1.48]	
Histologic grade				
I–II	Ref.	< 0.001^†^	Ref.	0.003
III	2.25 [1.60–3.16]		1.91 [1.25–2.94]	
Lymphovascular invasion				
Present	Ref.	0.001^†^	Ref.	0.001
Absent	0.57 [0.40–0.80]		0.51 [0.35–0.75]	
Hormone receptor status				
Positive	Ref.	< 0.001^†^	Ref.	0.140
Negative	2.20 [1.58–3.08]		2.71 [0.72–10.22]	
HER2 receptor status				
Positive	Ref.	< 0.001^†^	Ref.	0.063
Negative	0.52 [0.35–0.75]		0.58 [0.33–1.03]	
Ki-67 index				
< 10%	Ref.	0.001^†^	Ref.	0.721
≧ 10%	1.76 [1.25–2.48]		1.08 [0.72–1.61]	
Neoadjuvant CTx				
Administered	Ref.	< 0.001^†^	Ref.	0.244
Not administered	0.49 [0.34–0.72]		0.70 [0.39–1.27]	
Adjuvant CTx				
Administered	Ref.	0.819	Ref.	0.008
Not administered	0.96 [0.68–1.36]		1.78 [1.17–2.73]	
Adjuvant RTx				
Administered	Ref.	< 0.001^†^	Ref.	< 0.001
Not administered	3.84 [2.43–6.05]		5.74 [3.52–9.34]	
Adjuvant HTx				
Administered	Ref.	< 0.001^†^	Ref.	0.520
Not administered	2.22 [1.59–3.10]		0.65 [0.17–2.42]	
HER2-targeted treatment				
Administered	Ref.	0.095	Ref.	0.251
Not administered	0.69 [0.43–1.09]		1.50 [0.75–2.99]	
Operation method				
Conventional BCS	Ref.	0.004^†^	Ref.	0.036
Central lumpectomy	2.95 [1.38–6.32]		2.65 [1.07–6.60]	

*CI* confidence interval, *HER2* human epidermal growth factor receptor-2, *CTx* chemotherapy, *RTx* radiotherapy, *HTx* hormone treatment, *BCS* breast-conserving surgery.

^*^Stratified according to the American Joint Committee on Cancer (AJCC) 7th TNM stage, patients who underwent neoadjuvant CTx were evaluated with clinical stage

^†^Values were calculated for Cox regression analysis

Additionally, we conducted PSM yielding 99 and 297 patients in the CL and conventional BCS groups, respectively (Table 3). After matching, IBTR events were observed in five cases each in both the CL (5.1%) and conventional BCS (1.7%) groups. Similar to the comparison of the original unmatched cohorts, the CL group was associated with a significantly higher IBTR than the conventional BCS group on log-rank test (HR, 3.27; 95%CI, 0.94–11.36, log-rank *p* = 0.048) (Fig. 1B), while the recurrence-free and OS rates were also comparable between the two groups (Supplementary Fig. S3). Lastly, we conducted IPTW to further reduce selection bias by giving more weights to less common, but potentially more informative population. The effective sample size was the same as the original cohort, but weighted survival analysis still showed a significantly higher IBTR for the CL group compared to the conventional BCS group (HR, 4.66; 95%CI, 1.85–11.77; *p* < 0.001) (Supplementary Fig. S4).

**Table 3:** Clinical characteristics of patients according to the operation method after propensity score matching

Characteristics	Central lumpectomy (*n* = 99)	Conventional BCS (*n* = 297)	*p*-value
Age at operation (years)^*^	51.0 ± 9.1	51.6 ± 9.9	0.573
< 50	42 (42.4%)	117 (39.4%)	0.594
≧ 50	57 (57.6%)	180 (60.6%)	
Year of surgery			
2004–2012	35 (35.4%)	91 (30.6%)	0.383
2013–2018	64 (64.6%)	206 (69.4%)	
T stage^†^			
T1	61 (61.6%)	182 (61.3%)	0.830
T2	35 (35.4%)	109 (36.7%)	
T3–4	3 (3.0%)	6 (2.0%)	
N stage^†^			
N0	60 (60.6%)	183 (61.6%)	0.391
N1	32 (32.3%)	81 (27.3%)	
N2–3	7 (7.1%)	33 (11.1%)	
Histologic grade			
I–II	60 (60.6%)	190 (64.0%)	0.548
III	39 (39.4%)	107 (36.0%)	
Lymphovascular invasion			
Present	36 (36.4%)	100 (33.7%)	0.625
Absent	63 (63.6%)	197 (66.3%)	
Hormone receptor status			
Positive	81 (81.8%)	240 (80.8%)	0.824
Negative	18 (18.2%)	57 (19.2%)	
HER2 receptor status			
Positive	27 (27.3%)	71 (23.9%)	0.501
Negative	72 (72.7%)	226 (76.1%)	
Ki-67 index			
< 10%	70 (70.7%)	207 (69.7%)	0.849
≧ 10%	29 (29.3%)	90 (30.3%)	
Neoadjuvant CTx			
Administered	14 (14.1%)	46 (15.5%)	0.746
Not administered	85 (85.9%)	251 (84.5%)	
Adjuvant CTx			
Administered	57 (57.6%)	175 (58.9%)	0.814
Not administered	42 (42.4%)	122 (41.1%)	
Adjuvant RTx			
Administered	90 (90.9%)	268 (90.2%)	0.844
Not administered	9 (9.1%)	29 (9.8%)	
Adjuvant HTx			
Administered	77 (77.8%)	231 (77.8%)	1.000
Not administered	22 (22.2%)	66 (22.2%)	
HER2-targeted treatment			
Administered	18 (18.2%)	45 (15.2%)	0.475
Not administered	81 (81.8%)	252 (84.8%)	

*BCS* breast-conserving surgery, *HER2* human epidermal growth factor receptor-2, *CTx* chemotherapy, *RTx* radiotherapy, *HTx* hormone treatment.

^*^Values are means ± standard deviation

^†^Stratified according to the American Joint Committee on Cancer (AJCC) 7th TNM stage, patients who underwent neoadjuvant CTx were evaluated with clinical stage

### Survival analysis for tumors located near the NAC

**Fig. 2:**
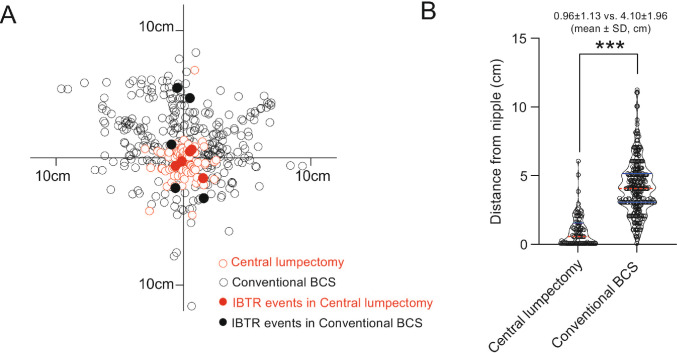
Location of the tumors relative to the nipple following propensity score matching. Following 1:3 propensity score matching, the tumor locations of 396 patients relative to the nipple are shown. Tumors associated with IBTR during follow-up are shown as filled circles (A). The distance from the nipple was significantly shorter in the CL group than in the conventional BCS group (B). *BCS* breast-conserving surgery, *SD* standard deviation, *IBTR* ipsilateral breast tumor recurrence

**Fig. 3:**
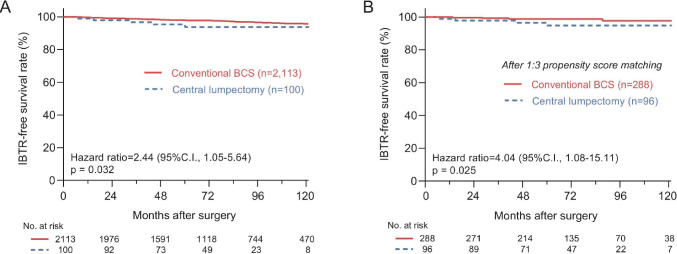
Kaplan–Meier curves for patients with tumor location within 3 cm from the nipple. Kaplan**–**Meier curves show the differences in IBTR between the two groups before (A) and after (B) 1:3 propensity score matching. P-values were calculated using the log-rank test and hazard ratios were calculated using the Cox regression test. *IBTR* ipsilateral breast tumor recurrence, *BCS* breast-conserving surgery, *CI* confidence interval

Because tumors in the CL group were located more centrally near the nipple, the distance between the NAC and tumors was significantly shorter than that in the conventional BCS group (*p* < 0.001, Fig. 2). To determine the impact of tumor-nipple distance on survival outcomes, we selected tumors located within 3 cm from the nipple on preoperative MRI or breast sonography, yielding 100 and 2,113 patients in the CL and conventional BCS groups, respectively. After this selection, the CL group remained associated with higher IBTR than the conventional BCS group (HR, 2.44; 95%CI, 1.05–5.64, log-rank *p* = 0.032) (Fig. 3A). Furthermore, we conducted a 1:3 PSM for these patients (Supplementary table S1). Among the 96 and 288 patients in each group, the CL group continued to show higher IBTR than the conventional BCS group (HR, 4.04; 95%CI, 1.08–15.11, log-rank *p* = 0.025) (Fig. 3B).

### Recurrence pattern of IBTR after surgery

The pattern of IBTR for those who underwent conventional BCS showed a double-peaked pattern, with the first peak at year 2 and the second peak between years 8 and 9 after surgery, which was similar to our previous report on locoregional recurrence patterns [12]. On contrast, the pattern of IBTR for the CL group showed significantly higher incidence until the first 6 years of surgery, but dramatically decreased thereafter. (Supplementary Fig. S5).

## Discussion

In the current study, we found CL to be associated with significantly higher IBTR compared to conventional BCS before and after PSM. The results were consistent for tumors confined to 3 cm from the nipple. In contrast, there was no significant difference in regional and distant recurrence, as well as OS, based on the surgical method.

Mastectomy is frequently favored for CLBCs due to concern for oncologic safety and apprehensions regarding unsatisfactory cosmetic outcomes following CL [13]. Recent studies, however, have reported the safety of BCS for CLBC and introduced several surgical techniques [6, 7, 14, 15]. In addition, with the development of NAC reconstruction methods, the disadvantages of CL can be reduced [16]. Considering that BCS can achieve higher cosmetic satisfaction and quality of life than total mastectomy, the oncologic safety of CL warranted studying [3, 4]. Few studies, however, have investigated locoregional recurrence following CL. Our findings suggest that although CL can be regarded as oncologically safe, post-operative surveillance of IBTR should be performed.

Several reports have studied the survival of patients undergoing BCS for CLBC. Simmons et al. retrospectively analyzed 99 patients and reported that the local or distant recurrence rate of central breast cancer did not differ significantly between lumpectomy and mastectomy at a median follow-up of 33 months (local recurrence, 6.3% vs. 3.5%; distant recurrence, 3.1% vs. 3.0; both *p* > 0.99)[17]. Another retrospective study which analyzed 333 patients compared BCS with and without nipple resection and showed comparable breast-free survival rates between the two groups [18]. These findings were supported by two SEER data studies [7, 19]. Liu et al. analyzed 8,702 patients (3,870 and 4,832 patients in the BCS (including CL) and non-BCS group, respectively) with CLBC and showed that the BCS group had a higher breast cancer-specific survival rate (*p* < 0.001) and OS rate (*p* < 0.001) than the non-BCS group. Fitzal et al. reviewed 1,485 patients (105, 1312, and 68 patients with CL for CLBC, mastectomy for CLBC, and conventional BCS for non-CLBC, respectively) and found no difference in OS (*p* = 0.348) and recurrence-free survival (*p* = 0.649) between CL and mastectomy for CLBC at a median follow-up of 35.3 months. Further, they failed to show a difference in local recurrence (*p* = 226) between the CL and conventional BCS groups. Unlike previous studies, we found a higher local recurrence rate following CL. This may be because previous studies only included patients with stage I and II breast cancer and excluded patients who underwent neoadjuvant chemotherapy. Our study included patients with stage III disease and those who received neoadjuvant chemotherapy. Additionally, adjustment for other variables with Cox regression analysis, PSM, and IPTW, along with a longer follow-up period, supports our results.

CL is mainly performed for subareolar tumors. Breast cancers originating from the major lactiferous ducts are reported to have different clinical, histopathological, and mammographic presentations compared to tumors originating from the terminal ductal and lobular units (TDLU) [20]. In the major ducts, cancer cells distend and distort normal ductal structures and often form new duct-like structures with massive tumor burdens. Tabar et al. reported that the 24-year cumulative survival of invasive breast cancer patients with both TDLUs and the main ductal component involvement was poorer than patients with tumors originating from the TDLU portion only (RR, 9.04; 95%CI, 4.78–17.08) [21]. Anatomically, the major lactiferous ducts converge into the nipple, and there is a higher risk for tumors close to the nipple to invade the major ducts. These findings suggest that tumors in the central portion of the breast may behave differently compared to tumors originating from other parts of the breast, resulting in different survival outcomes.

Furthermore, the observation that recurrent tumors in the CL group of the current study were not localized solely around the nipple, and that the distance between the recurrent tumor and the nipple was not significantly different between the two groups, provides further support for this theory. (Supplementary Fig. S6).

Breast tumor location may be an independent prognostic factor for survival outcomes [21, 22]. According to published studies, the prognosis of breast cancer varies depending on the tumor location in the breast, with the best prognosis for tumors in the upper outer quadrant. However, central breast cancers have limited data. Ji et al. reported greater disease severity and poorer survival of patients with tumors in the central and nipple portions [23]. They showed that tumors located in the central and nipple portion were associated with older age, larger tumor size, advanced tumor stage, and axillary node metastasis, which resulted in poorer breast cancer-specific survival (*p* = 0.005) and OS (*p* < 0.0001). Accordingly, we sub-analyzed tumors located 3 cm from the NAC in an effort to nullify the effect of distance to the nipple on survival outcome. The results showed patients undergoing CL to have a higher IBTR despite PSM.

The current study had several limitations. First, this retrospective study from a single institution may be associated with selection bias. Other variables affecting IBTR events such as the administration of radiation boost and multifocal lesions were not investigated. However, most of the patients underwent radiotherapy and achieved a clear resection margin in pathology reports, which are the strongest predictors of IBTR. Small number of patients in the CL group compared to majority of patients in the conventional BCS group is a mismatched comparison and requires caution in interpretation, notwithstanding our sensitivity analyses including multivariate analysis and PSM resulted in consistent result. Another limitation is subjectivity in the process leading to CL. CL was considered when the tumor was located close to the nipple and when nipple excision was deemed inevitable. Retrospective analysis on the location of the tumors showed that most of the tumors in the CL group were located within 3 cm from the nipple. However, there was no strict criteria for “close” distance when making the choice for surgical technique, and it heavily relied on the surgeon’s discretion and size of the breast. Due to these limitations, our current results warrant careful interpretation and multi-institutional investigations with a larger number of heterogeneous patients are needed to strongly support our findings. Finally, despite our PSM effort, we could not completely nullify the effect of the distance from the nipple, and the distance remained shorter in the CL group (Supplementary Fig. S7). Therefore, our study emphasizes on the comparison of surgical technique, rather than examining tumors only located under or close to the NAC area.

The absolute difference in IBTR between the CL group and the conventional BCS group was small, although it was statistically significant. Therefore, in the absence of any other differences in outcome, it would not be justified to perform mastectomy, alter RT dose or extent, or administer additional systemic therapy for patients who are eligible to receive CL.

In conclusion, patients who undergo CL have comparable DMFS and OS, but higher IBTR than those undergoing conventional BCS. For patients with CLBC, higher risk for IBTR associated with breast conservation should be informed before performing CL, and careful surveillance may be necessary during the early post-operative follow-up periods.

### Supplementary Information

Below is the link to the electronic supplementary material.

Supplementary file1 (DOCX 3730 kb)

## Data Availability

Ji-Jung Jung and Jong-Ho Cheun had full access to all the data in the study and takes responsibility for the integrity of the data and the accuracy of the data analysis. The datasets analyzed during the current study are not publicly available due to institutional regulations, but are available from the corresponding author on reasonable request.
