# Supplementary material for: Comparison of long-term oncologic outcomes of central lumpectomy and conventional breast-conserving surgery for invasive breast cancer: propensity score matching analysis
Source: Breast Cancer Res Treat. 2024 Mar 25;205(3):465–74. doi: 10.1007/s10549-024-07297-8 (PMC11101516; doi:10.1007/s10549-024-07297-8)

**eSupplemental material**

**Comparison of long-term oncologic outcomes of central lumpectomy and conventional breast-conserving surgery for invasive breast cancer**

**Table of contents**

Surgical approach for central lumpectomy Fig. S1

Kaplan–Meier curves comparing the surgical methods Fig. S2

Kaplan–Meier curves comparing the surgical methods following propensity score matching. Fig. S3

Kaplan–Meier curve for ipsilateral breast tumor recurrence free survival following inverse probability of treatment weighting analysis. Fig. S4

Clinical characteristics of patients whose tumors were located within 3.0cm from the nipple
after 1:3 propensity score matching Table. S1

IBTR pattern after surgery Fig. S5

Location of recurred tumors relative to the nipple for all patients Fig. S6

Tumor location within 3 cm from the nipple after 1:3 propensity score matching Fig. S7

**Figure S1. Surgical approach for central lumpectomy**

Patients who underwent central lumpectomy were approached through an elliptical incision (A) or circumareolar incision (B). Those tumors were located mainly close to the nipple (C)

**Figure S2. Kaplan–Meier curves comparing the surgical methods**


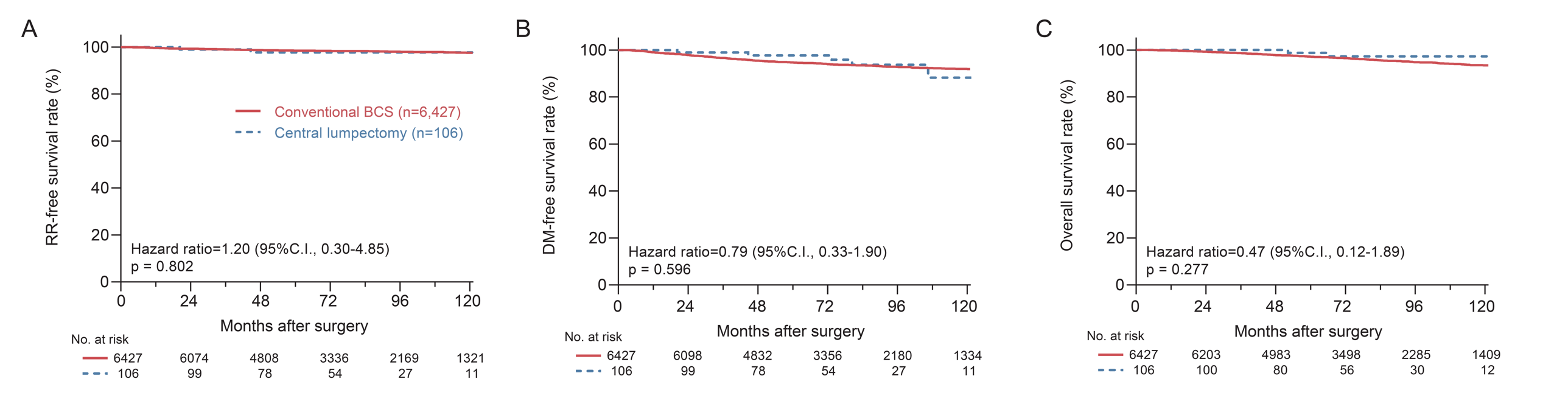


The Kaplan–Meier curves show the differences in regional recurrence (A), distant metastasis (B), and OS (C) between the two groups classified according to the surgical method. P-values were calculated using the log-rank test and hazard ratios were calculated using the Cox regression test.

Abbreviations: BCS, breast-conserving surgery; CI, confidence interval; RR, regional recurrence; DM, distant metastasis; OS, overall survival

**Figure S3. Kaplan–Meier curves comparing the surgical methods following propensity score matching**


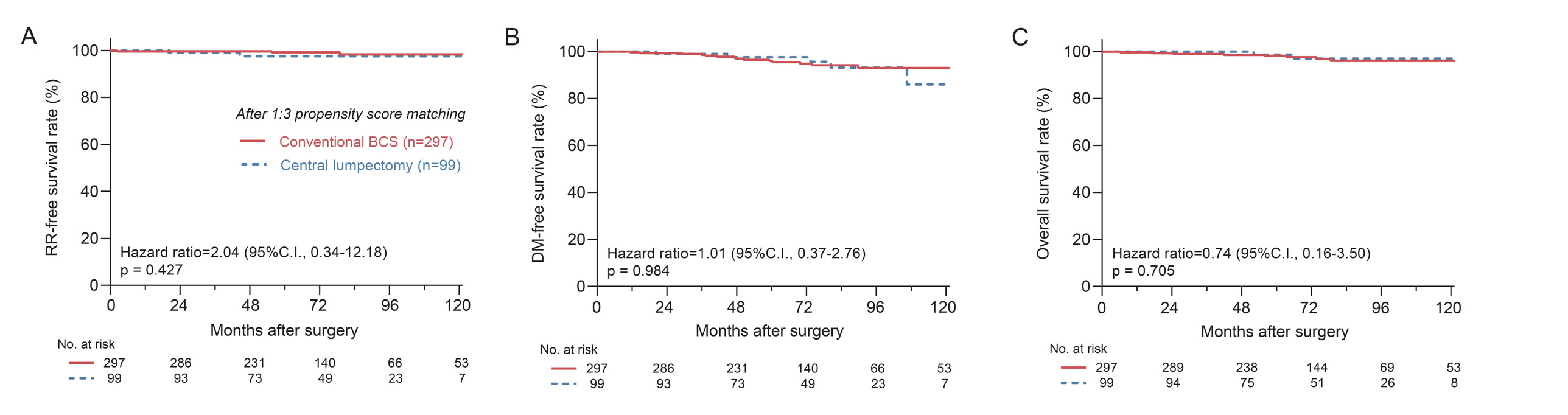


The Kaplan–Meier curves show the differences in regional recurrence (A), distant metastasis (B), and OS (C) between the two groups after 1:3 propensity score matching. P-values were calculated using the log-rank test and hazard ratios were calculated using the Cox regression test.

Abbreviations: BCS, breast-conserving surgery; CI, confidence interval; RR, regional recurrence; DM, distant metastasis; OS,

**Figure S4. Kaplan–Meier curve for ipsilateral breast tumor recurrence free survival following inverse probability of treatment weighting analysis**

**
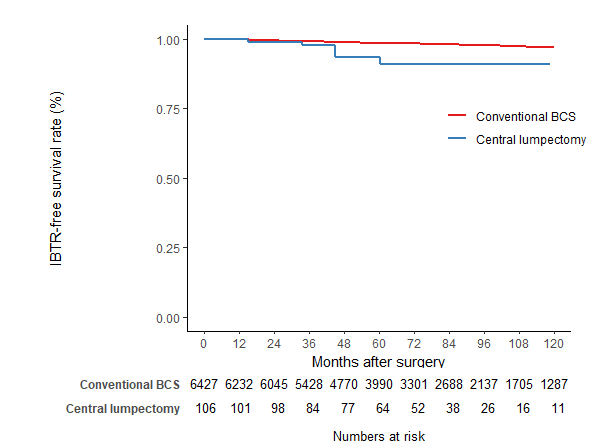
**

The Kaplan–Meier curve show the differences in IBTR between the two groups after inverse probability of treatment weighting analysis. P-values were calculated using the log-rank test and hazard ratios were calculated using the Cox regression test.

Abbreviations: BCS, breast-conserving surgery; CI, confidence interval; IBTR, ipsilateral breast tumor recurrence.

**Table S1. Clinical characteristics of patients whose tumors were located within 3.0cm from the nipple after 1:3 propensity score matching**

| Characteristics | Central lumpectomy (n=96) | Conventional BCS (n=288) | *p*-value |
| --- | --- | --- | --- |
| Age at operation (years) ^*^ | 50.7 ± 9.0 | 51.5 ± 9.8 | 0.472 |
| < 50 | 41 (42.7%) | 109 (37.8%) | 0.398 |
| ≧ 50 | 55 (57.3%) | 179 (62.2%) |  |
| Year of Surgery |  |  |  |
| 2004 – 2012 | 35 (36.5%) | 97 (33.7%) | 0.620 |
| 2013 – 2018 | 61 (63.5%) | 191 (66.3%) |  |
| T stage^†^ |  |  |  |
| T1 | 59 (61.5%) | 171 (59.4%) | 0.623 |
| T2 | 34 (35.4%) | 112 (38.9%) |  |
| T3-4 | 3 (3.1%) | 5 (1.7%) |  |
| N stage^†^ |  |  |  |
| N0 | 57 (59.4%) | 165 (57.3%) | 0.409 |
| N1 | 32 (33.3%) | 88 (30.6%) |  |
| N2-3 | 7 (7.3%) | 35 (12.2%) |  |
| Histologic grade |  |  |  |
| I-II | 60 (60.4%) | 184 (63.9%) | 0.542 |
| III | 38 (39.6%) | 104 (36.1%) |  |
| Lymphovascular invasion |  |  |  |
| Present | 36 (37.5%) | 100 (34.7%) | 0.622 |
| Absent | 60 (62.5%) | 188 (65.3%) |  |
| Hormone receptor status |  |  |  |
| Positive | 78 (81.3%) | 248 (86.1%) | 0.249 |
| Negative | 18 (18.8%) | 40 (13.9%) |  |
| HER2 receptor status |  |  |  |
| Positive | 27 (28.1%) | 70 (24.3%) | 0.456 |
| Negative | 69 (71.9%) | 218 (75.7%) |  |
| Ki-67 index |  |  |  |
| < 10% | 68 (70.8%) | 211 (73.3%) | 0.644 |
| ≧ 10% | 28 (29.2%) | 77 (26.7%) |  |
| Neoadjuvant CTx. |  |  |  |
| Administered | 14 (14.6%) | 49 (17.0%) | 0.578 |
| Not administered | 82 (85.4%) | 239 (83.0%) |  |
| Adjuvant CTx. |  |  |  |
| Administered | 56 (58.3%) | 164 (56.9%) | 0.812 |
| Not administered | 40 (41.7%) | 124 (43.1%) |  |
| Adjuvant RTx. |  |  |  |
| Administered | 88 (91.7%) | 269 (93.4%) | 0.564 |
| Not administered | 8 (8.3%) | 19 (6.6%) |  |
| Adjuvant HTx. |  |  |  |
| Administered | 75 (78.1%) | 243 (84.4%) | 0.160 |
| Not administered | 21 (21.9%) | 45 (15.6%) |  |
| HER2 targeted treatment |  |  |  |
| Administered | 18 (18.8%) | 48 (16.7%) | 0.639 |
| Not administered | 78 (81.3%) | 240 (83.3%) |  |

^*^Values are means ± standard deviation

^†^Stratified according to the American Joint Committee on Cancer (AJCC) 7^th^ TNM stage, patients who underwent neoadjuvant CTx. were evaluated with clinical stage

Abbreviations: BCS, breast-conserving surgery; HER2, human epidermal growth factor receptor-2; CTx., chemotherapy; RTx., radiotherapy; HTx, hormone treatment

**Figure S5. Recurrence pattern of IBTR after surgery**


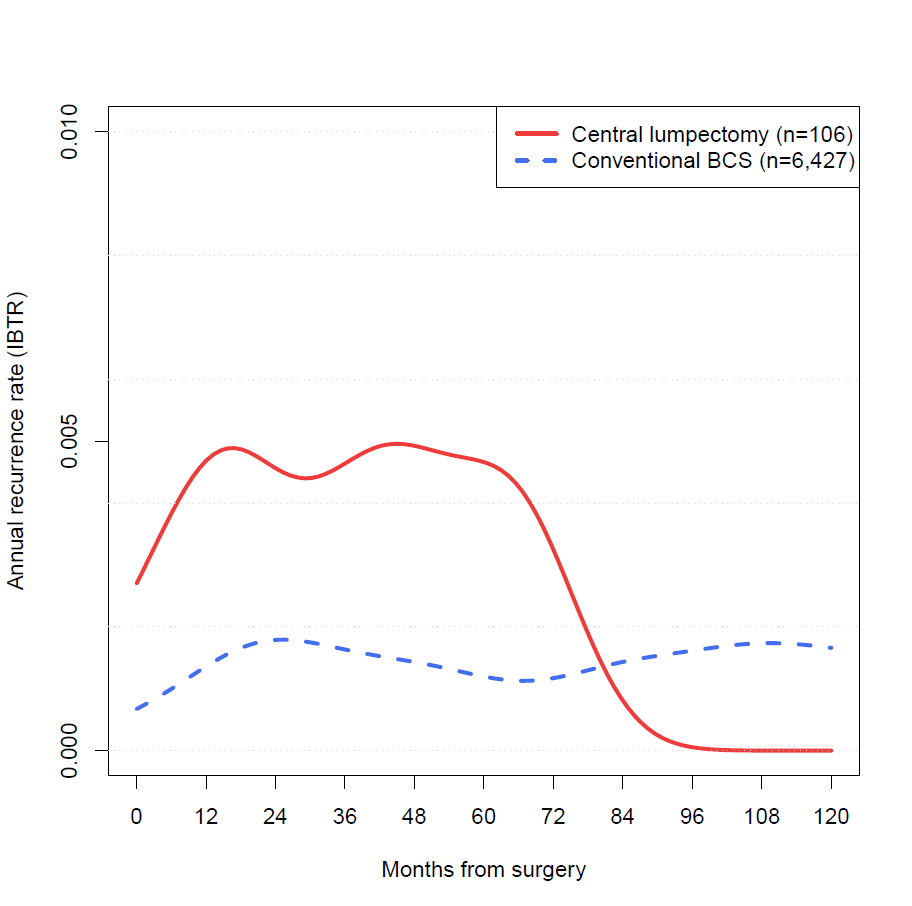


The pattern of IBTR according to the surgical method. The conventional BCS group has a double-peaked pattern, with the first peak at year 2 and the second peak between years 8 and 9 after surgery. The CL group shows higher incidence of IBTR in the first 6 years of surgery but lower afterwards.

Abbreviations: BCS, breast-conserving surgery; IBTR, ipsilateral breast tumor recurrence

**Figure S6. Location of recurred tumors relative to the nipple for all patients**

Locations of the recurred tumors relative to the nipple are shown (A) The distance between recurred tumor and nipple was not significantly different between the central lumpectomy group and conventional BCS group.

Abbreviations: BCS, breast conserving surgery; SD, stand deviation


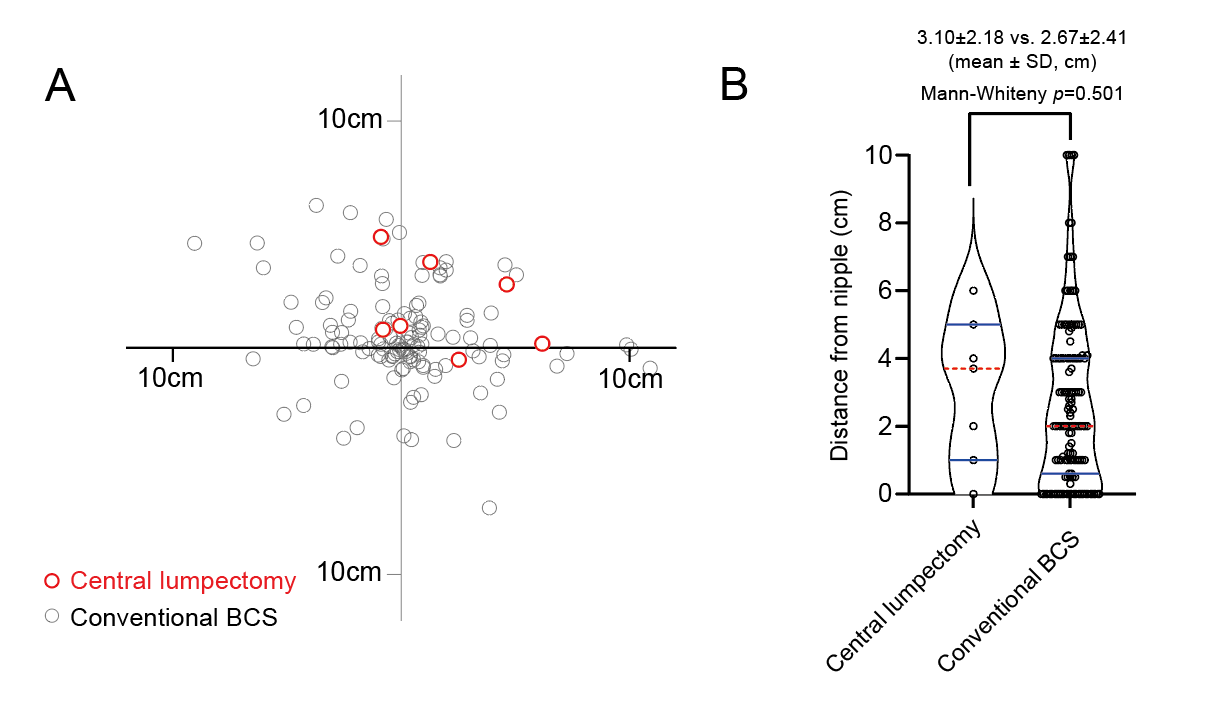


**Figure S7. Tumor location within 3 cm from the nipple after 1:3 propensity score matching**

After 1:3 propensity score matching, tumor locations relative to the nipple of 384 patients with tumors within 3 cm of the nipple are shown and tumors that show IBTR event during the surveillance are shown as filled circles (A). Distance from the nipple was significantly shorter for central lumpectomy group than the conventional BCS group (B).

Abbreviations: BCS, breast conserving surgery; SD, standard deviation; IBTR, ipsilateral breast tumor recurrence


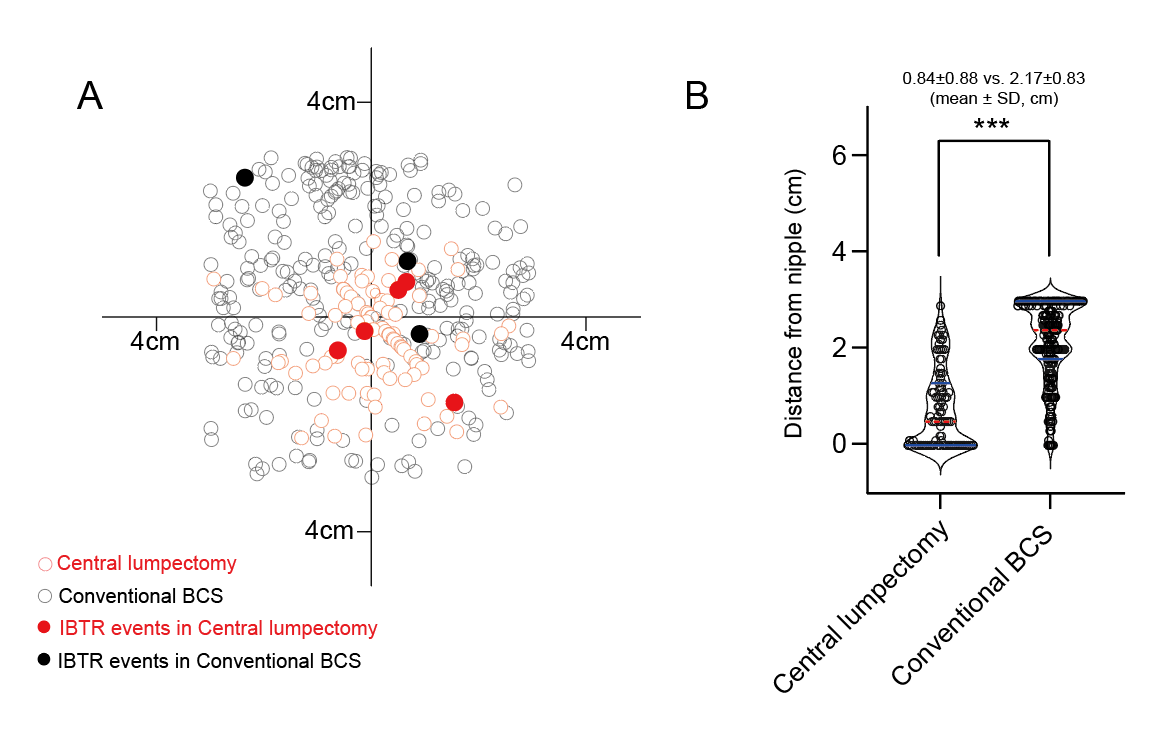

Supplement: Supplementary file 1 — Supplementary file1 (DOCX 3730 kb) [file 10549_2024_7297_MOESM1_ESM.docx]
